# Patient-Centric Mobile Medical Services Accessed Through Smartphones in the Top 100 Chinese Public Hospitals: Cross-Sectional Survey Study

**DOI:** 10.2196/45763

**Published:** 2024-12-04

**Authors:** Xuan Huang, Ying Wang, Xixian Yang, Ruo Jiang, Yicheng Liu, Hui Wang

**Affiliations:** 1 Department of Otolaryngology-Head and Neck Surgery Shanghai Sixth People's Hospital Affiliated to Shanghai Jiao Tong University School of Medicine Shanghai China; 2 Otolaryngology Institute of Shanghai Jiao Tong University Shanghai China; 3 Shanghai Key Laboratory of Sleep Disordered Breathing Shanghai China; 4 Shanghai Sixth People's Hospital Affiliated to Shanghai Jiao Tong University School of Medicine Shanghai China

**Keywords:** mobile health technology, smartphones, mobile phone, internet hospital, China

## Abstract

**Background:**

Smartphone-based technology has been used to enhance the delivery of health care services to the public in numerous countries.

**Objective:**

This study aims to investigate the application of patient-centric mobile medical services accessed through smartphones in the top 100 Chinese public hospitals.

**Methods:**

Data on 124 tertiary public hospitals, ranked among the top 100 by the China Hospital Science and Technology Evaluation Metrics of the Chinese Academy of Medical Sciences (2019) and China’s Hospital Rankings of the Hospital Management Institute of Fudan University (2019), were collected from the WeChat platform (Tencent Inc), mobile phone apps, and official websites until February 10, 2021.

**Results:**

A total of 124 tertiary public hospitals, all of which were among the top 100 hospitals according to the 2 ranking lists, were selected for this study. Almost all (122/124, 98.39%) of the hospitals offered basic services such as appointment scheduling, registration, and health education. The majority also provided online access to test reports (95/124, 76.61%), consultations (72/124, 58.06%), and prescriptions (61/124, 49.19%). Among the hospitals offering online prescriptions, the majority (54/61, 88.52%) supported home delivery through third-party carriers. Slightly less than half (57/124, 45.97%) used artificial intelligence for medical guidance. Only a small fraction (8/124, 6.45%) managed chronic diseases through online monitoring and supervision by experienced doctors. Approximately half (60/124, 48.39%) of the included hospitals were officially licensed as internet hospitals approved to provide full online services. Hospitals with official internet hospital licenses provided more extensive digital health offerings. A significantly higher proportion of approved hospitals offered online consultations (29.69% vs 88.33%, *r*=43.741; *P*<.001), test reports (62.5% vs 91.67%, *r*=14.703; *P*<.001), and chronic disease management (1.56% vs 11.67%, *r*=5.238; *P*<.05). These officially approved hospitals tended to provide over 6 mobile medical services, mainly in the regions of Shanghai and Guangdong. This geographic distribution aligned with the overall layout of hospitals included in the study.

**Conclusions:**

Patient-centric mobile medical services offered by the top 100 Chinese public hospitals accessed through smartphones primarily focus on online appointment scheduling, registration, health education, and accessing test reports. The most popular features include online consultations, prescriptions, medication delivery, medical guidance, and early-stage chronic disease management. Approved internet hospitals offer a significantly greater variety of patient-centric mobile medical services compared with unapproved ones.

## Introduction

Mobile medical services represent a contemporary concept, encompassing the use of wireless technology to deliver health services and information through mobile communication devices [1-3]. With the advent of mobile communications supported by 4G and 5G mobile networks, mobile medical services have the potential to offer health care services at any time and place, overcoming geographical and temporal barriers. Such offerings alleviate patient expenses and the inconvenience of traveling to major cities for specialized expertise [4]. In the past few years, mobile medical services have experienced exponential growth, propelled by the rapid expansion of the internet and the increasing prevalence of the internet and mobile devices such as mobile phones or smartphones [5,6]. Smartphones, ubiquitous as personal computers today, have revolutionized the communication landscape, with more than 95% of Chinese adults owning a mobile phone [7]. Such high coverage of mobile access provides an unprecedented opportunity for mobile-based health services. These devices, nearly always active and highly portable, accompany users wherever they go, facilitating real-time, on-demand communication and a wide array of service functions. High-quality, robust medical care and health system services can be fulfilled by approved internet hospitals by using mobile technology [8].

Internet hospitals have emerged as a significant trend in China in recent years due to their potential to provide widely accessible patient service delivery [9-11]. An internet hospital refers to a facility capable of delivering outpatient services through internet technologies, allowing patients to consult with doctors or manage chronic diseases through websites, mobile medical services apps, or WeChat platforms in Chinese public hospitals. Patients can receive medical consultations and send images of auxiliary examinations to the doctor through the internet. Meanwhile, the doctor can inquire about the patient’s health status and offer advice. Some of these hospitals have been authorized with Internet Hospital licenses by the local government so that they can provide additional services like online prescriptions and direct payment through government medical insurance. These hospitals are referred to as approved internet hospitals. The first officially approved Internet Hospital was launched on Oct 25, 2014, in Guangdong Province, China. Mobile medical services provided by internet hospitals have greatly diminished geographical and time constraints, thereby offering Chinese patients online access to skilled doctors and addressing the issue of long wait times [12]. Many public hospitals now offer online personal health records, enabling patients to access their own health information, refill prescriptions, schedule appointments, receive online consultations and remote diagnosis, obtain electronic prescriptions, access other health care management supports, review diagnoses or checkup reports, and make payments through mobile medical services applications (apps) or the WeChat platform [10]. The market value of Chinese internet medical services was 11.4 billion yuan (US $1.7 billion) in 2014 and 15.7 billion yuan in 2015, marking an increase of 37.7%. The user population of internet medical services users was 194.8 million in 2016, accounting for 26.6% of all internet users [11]. WeChat, launched in January 2011, is one of the world’s leading social networks, ranking fifth in terms of active users. As of 2020, it had become one of the world’s largest mobile apps, with over 1 billion monthly active users [13]. Mobile health care services represent a novel endeavor to provide patient-centric health care in China. Patient-centric health care is defined as care provision that is consistent with the values, needs, and desires of the patient. It is thought to offer numerous benefits, including better health outcomes, greater patient satisfaction, and reduced health costs [14,15]. Mobile health care services can significantly reduce patients’ health expenses by reducing transportation time and hospital waiting times. In addition, online chronic disease management allows patients to upload daily health data like weight and glucose levels, and they can receive updated instructions from doctors and nutritionists remotely. Instead of manually recording these metrics, mobile services can automatically generate graphs for easy comprehension, which will finally lead to improved health outcomes and heightened patient satisfaction. Especially from late 2019 to early 2020, an outbreak of COVID-19 swept throughout China [16]. Due to the risk of contracting COVID-19, patients are currently less inclined to visit offline clinics or hospitals. In addition, the procedure for visiting offline clinics is more complex because of the requirement for screening tests and answering questionnaires. Under such circumstances, approved internet hospitals offer numerous advantages in providing outpatient health care in China while adhering to the guidelines of the nationwide epidemic prevention and control program [17,18]. In return, internet hospitals can also help to control the COVID-19 epidemic. In terms of the COVID-19 features, rapid online reservations for nucleic acid testing and free COVID-19–related consultations are the main differentiating factors from other services. Patients who do not need regular medical consultations but only need nucleic acid testing for travel purposes, etc, can bypass many steps like registration. Furthermore, patients exhibiting abnormal body temperature, epidemic exposure history, or COVID-19–related symptoms like cough or malaise can have direct access to specialists who can give standard instructions and transfer these cases separately.

However, mobile medical programs by both approved internet hospitals and unapproved internet hospitals are still in the exploratory stage, and many problems remain to be solved, such as integrating internet-based medical services into health insurance programs, ensuring surveillance and quality control, assessing the applicability of internet diagnoses for certain diseases, addressing possible medical disputes, and evaluating long-term return on investment [10]. The proliferation of mobile medical services in China suggests a promising direction for future health care. However, to date, patient-centric mobile medical services accessed through smartphones across the country have not been systematically investigated.

The objective of this study was to provide an overview of mobile medical services accessed through smartphones in China among the top 100 hospitals ranked by the China Hospital Science and Technology Evaluation Metrics of the Chinese Academy of Medical Sciences (2019) and China’s Hospital Rankings of the Hospital Management Institute of Fudan University (2019). We conducted a cross-sectional study to describe the characteristics of China’s mobile medical services accessed through smartphones and to assess their health service capacities.

## Methods

### Data Collection

We conducted a cross-sectional survey to assess the use of smartphone-based patient-centric services in the top 100 hospitals listed in 2 separate ranking systems: the China Hospital Science and Technology Evaluation Metrics of the Chinese Academy of Medical Sciences (2019) and China’s Hospital Rankings of the Hospital Management Institute of Fudan University (2019).

The 2 lists ranked the top 100 hospitals for medical care all over China by 2 different assessment methodologies. The China Hospital Science and Technology Evaluation Metrics of the Chinese Academy of Medical Sciences (2019) was based on the concept of science and technology evaluation metrics (STEMs). It provides a comprehensive assessment covering all scientific and technological activities and the entire chain of innovative endeavors within hospitals, reflecting the breadth and depth of the impact of scientific and technological activities in the hospitals pertinent to 3 aspects: scientific and technological output, academic impact, and overall scientific and technological conditions according to unified standards, sources, and methodologies [19]. China’s Hospital Rankings of the Hospital Management Institute of Fudan University (2019) [20] primarily relied on reputation evaluation based on the organizational structure of hospital departments, clinical technology and medical quality, and the scientific research level across 37 specialized clinical departments.

After identifying 124 hospitals in total, we searched the official websites of each hospital to check whether they presented the official QR code of mobile platforms, including WeChat and mobile health services apps. Out of these, 123 hospitals provided either WeChat or mobile health services apps. Then, we used smartphones to search and subscribe to the platform of each hospital and tested and recorded the online health care services provided. An Excel (Microsoft Corp) spreadsheet was used to record this information from each hospital. As for these 124 hospitals, mobile medical services included outpatient appointment scheduling, online consultations, medical guidance systems, access to online test reports, online prescriptions, sale of prescription drugs, medication delivery services, online payment services, provision of chronic disease management, and health education information.

Finally, due to specific regulatory policies in some regions, certain health care services, including online descriptions and the sale of prescription drugs, can only be provided by hospitals authorized with internet hospital licenses. To assess the difference between approved internet hospitals and unapproved internet hospitals in the provision of smartphone-based patient-centric mobile medical services in China, we used Baidu, the largest Chinese search engine, to identify officially approved internet hospitals. Hospitals that had announced obtaining internet hospital licenses, as indicated by related news and reports, were included in this analysis up to February 10, 2021. We cross-referenced the search results through the hospitals’ official WeChat accounts and added any internet hospitals that were officially approved to this analysis. A total of 2 investigators performed the data collection independently.

### Recruitment

The China Hospital Science and Technology Evaluation Metrics by the Chinese Academy of Medical Sciences and China’s Hospital Rankings by the Hospital Management Institute of Fudan University were both publicly released. Data on the hospitals in these rankings were collected solely from their public WeChat official accounts and mobile health service apps accessed through smartphones. As a result, no individual recruitment was needed, and there were no specific studies or clinical settings for recruitment or direct recruitment procedures.

### Statistical Analysis

Data collection and analyses were performed using Microsoft Excel 2016. Categorical variables were compared using the chi-square test. All data analyses were performed using the IBM SPSS (version 22.0) predictive analytic software. The statistical map was drawn by DataFocus (DATAFOCUS.PTE.LTD).

### Ethical Considerations

In this study, data were collected exclusively from public WeChat official accounts and mobile health service apps of hospitals listed in the publicly released China Hospital Science and Technology Evaluation Metrics and China’s Hospital Rankings. The focus was solely on observing public behavior. To ensure privacy and confidentiality, no personal data were gathered, and therefore, institutional review board approval was not required. Furthermore, no compensation was provided to human subjects, and no images identifying individual participants were included in the article or supporting material.

## Results

A total of 124 tertiary public hospitals from the 2 ranking lists of the top 100 hospitals were included in this study. We identified 60 active approved Internet hospitals, mostly concentrated in Shanghai and Guangdong. Mobile medical services primarily included appointment scheduling and registration, online medical guidance systems and consultations, online prescriptions, medication delivery services, online test reports, online payments, chronic disease management, and health education etc (more details in Table 1). Overall, 98.39% (122/124) of the hospitals offered appointment scheduling and registration services and provided health education online; 76.61% (95/124) of the hospitals supplied online test reports; and 58.06% (72/124) of the hospitals provided online consultations. In addition, 49.19% (61/124) of the hospitals offered online prescriptions; among these hospitals, 88.52% (54/61) also provided home delivery services using third-party delivery companies. A total of 45.97% (57/124) of the hospitals launched medical guidance systems based on artificial intelligence technology. Only 6.45% (8/124) of the hospitals offered chronic disease management modules under the monitoring and management of experienced medical teams.

**Figure 1:**
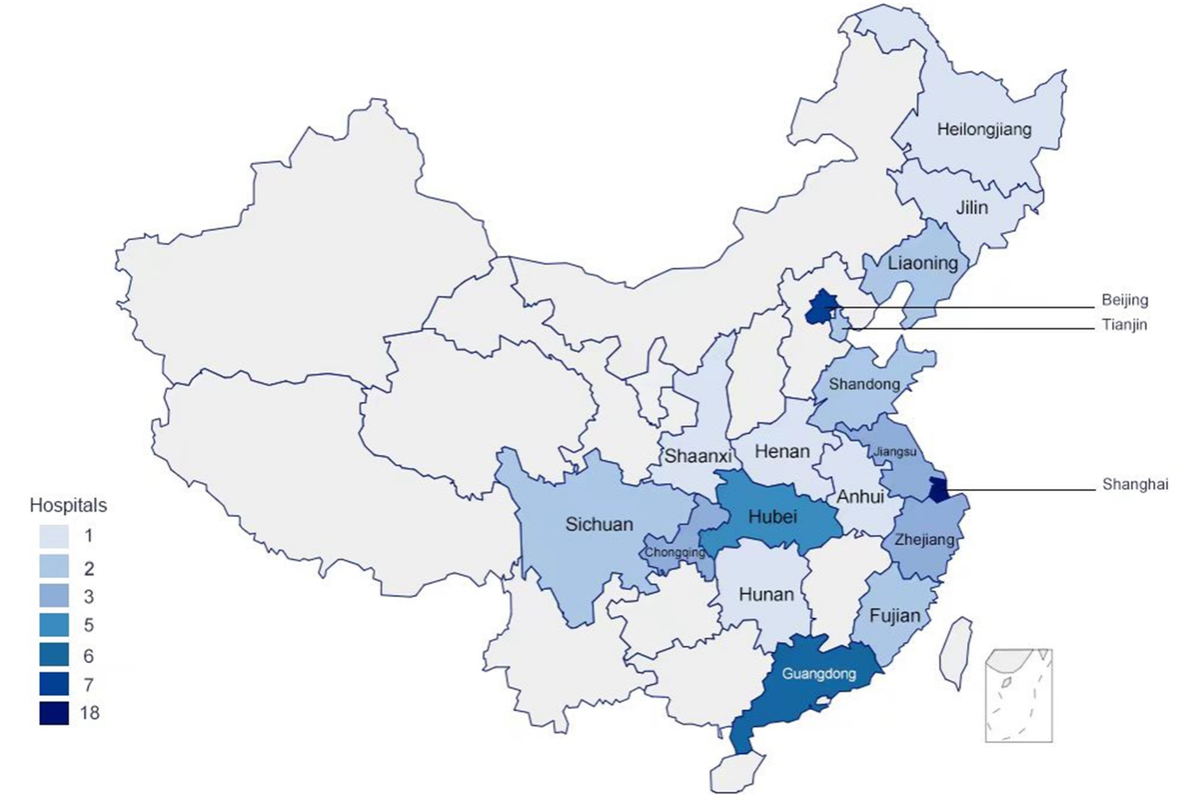
Geographic distribution of hospitals offering more than 6 mobile medical services accessible through smartphones among the top 100 ranked hospitals in China in 2021.

**Figure 2:**
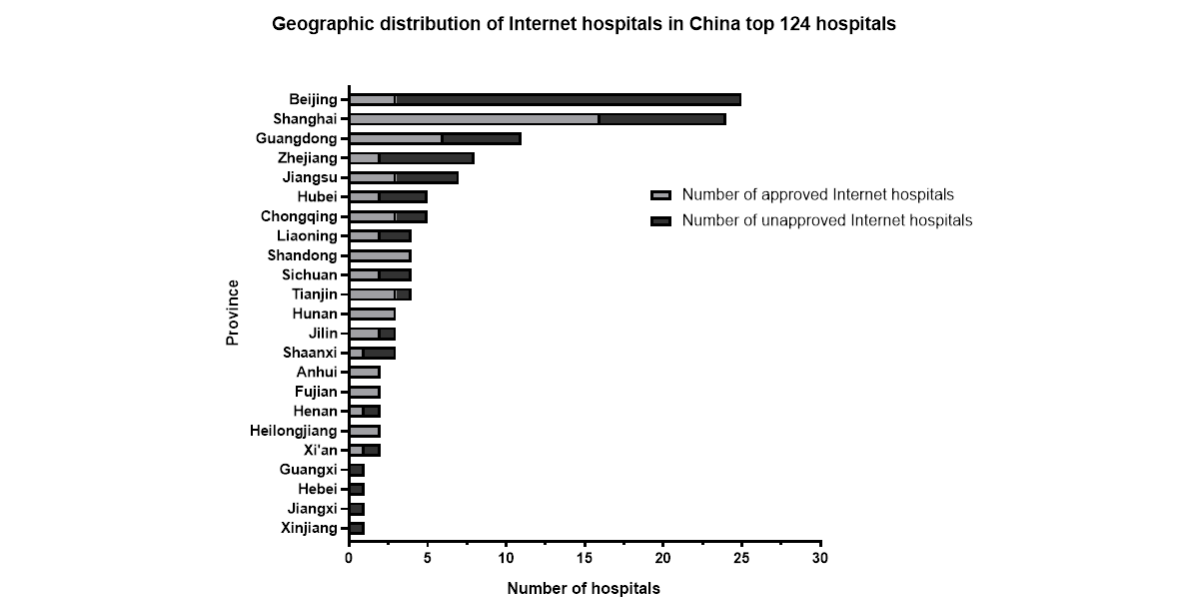
Distribution of approved and unapproved internet hospitals among the top 100 ranked hospitals in China categorized by province in 2021.

We mapped the geographic distribution of hospitals among the top 100 hospitals rankings that offered more than 6 smartphone-accessible mobile medical services (Figure 1). These hospitals were predominantly concentrated in Shanghai, Beijing, and Guangdong (Figure 2). Shanghai had the highest number, with 18 hospitals providing more than 6 mobile medical services, followed by Beijing with 7 and Guangdong with 6. The number of hospitals in other provinces ranged from 1 to 4, highlighting significant regional disparities in the availability of mobile medical services across China. Beijing housed 25 of the top 100 hospitals in China, the largest number among all provinces, yet it had only 3 approved internet hospitals. Conversely, Shanghai demonstrated a higher concentration of approved internet hospitals (Figure 2).

**Table 1:** Types of mobile medical services offered by approved internet hospitals and the percentage of these hospitals offering each type of service in 2021.

Types of mobile medical services	Number of hospitals offering each type of service (N=124), n	Prevalence across these hospitals, %
Appointment scheduling and registration services	122	98.39
Online health education	122	98.39
Online test reports	95	76.61
Online consultations	72	58.06
Online prescriptions	61	49.19
Home delivery services	54	88.52
Medical guidance system (artificial intelligence technology)	57	45.97
Chronic disease management	8	6.45

In addition, we sought to identify the differences in overall health care and mobile health care service apps for patient-centric services between approved internet hospitals and unapproved internet hospitals. The chi-square test revealed significant discrepancies in the availability of numerous vital functions between the 2 categories. Among the 124 hospitals examined, 48% (60/124) of the hospitals were registered internet hospitals. Compared with unapproved internet hospitals, a higher proportion of approved internet hospitals provided online consultation (29.69% vs 88.33%, *r*=43.741; *P*<.001), test reports (62.5% vs 91.67%, *r*=14.703; *P*<.001), and chronic disease management services (1.56% vs 11.67%, *r*=5.238; *P*<.05).

**Figure 3:**
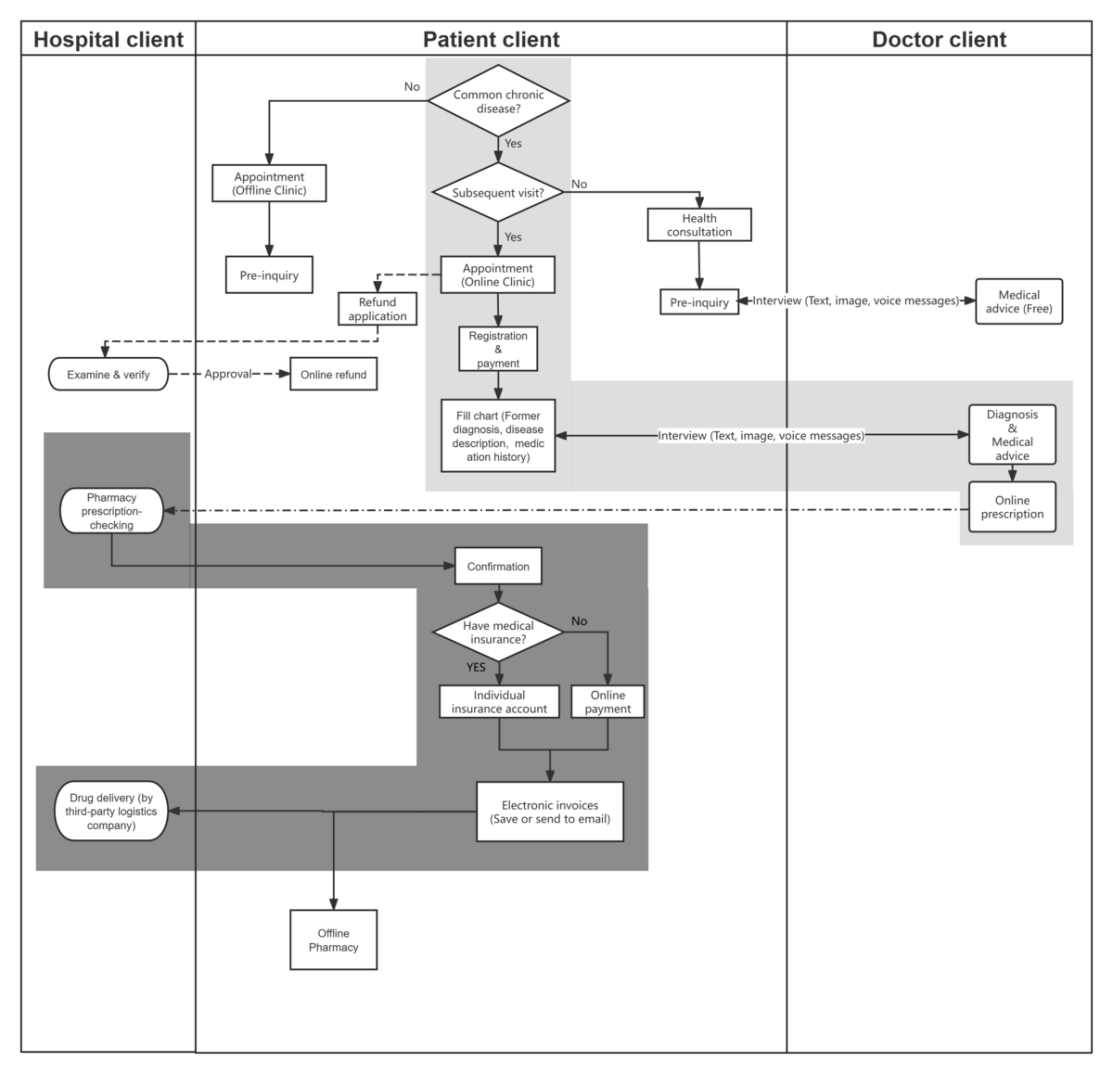
Flowchart illustrating the outpatient treatment processes in a typical internet hospital involved in the outpatient care process from the perspectives of the hospital, patient, and doctor client.

**Figure 4:**
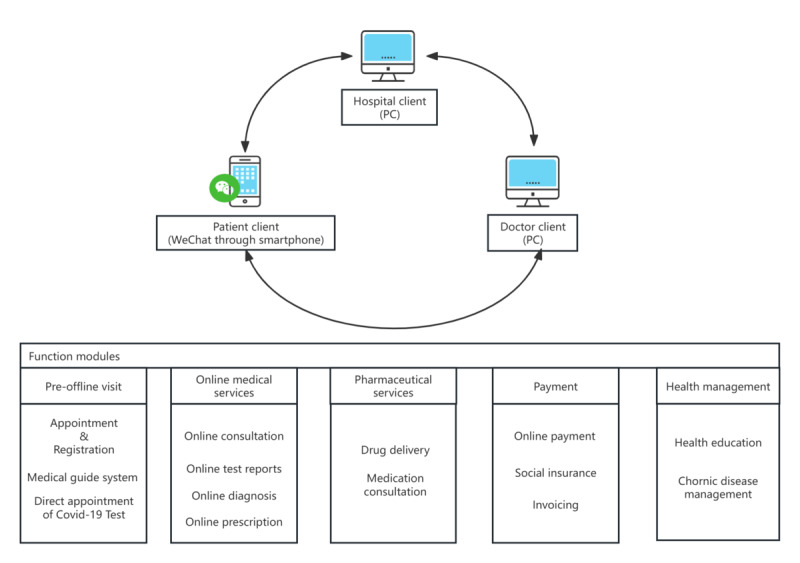
Service structure of a typical approved internet hospital from appointment scheduling to ongoing health management.

From Shanghai Sixth People’s Hospital, an approved internet hospital, we extracted the workflow of medical processes through a smartphone and drew a sequence diagram for this hospital function (Figure 3). Patients with common chronic diseases should first confirm that the hospital can provide medical services for that disease, which means that the disease is included in the list. Upon confirmation, patients can update their current status to doctors online instead of visiting the clinic. The light gray area in Figure 4 shows the process of an online visit by patients with common chronic diseases. Next, patients can choose a doctor online and schedule appointments for online consultations. Similar to the offline procedure, patients register on the appointment day, complete payment, and fill out basic charts detailing their concerns, previous diagnoses, and medication history. Doctors diagnose and provide medical advice after the appointment through text, images, and voice messages, with the option to offer online prescriptions directly. The dark gray area in Figure 4 illustrates the process of how patients receive treatments and purchase prescription drugs online. After receiving the online prescriptions from the doctor, pharmacists handle the electronic prescriptions. Then, patients confirm the prescriptions and make payments online. In certain approved internet hospitals, patients with health insurance can use their insurance account after registration, after which prescribed drugs can be delivered home by a third-party logistics company cooperating with the hospital. Alternatively, drugs can be picked up at the hospital pharmacy. Patients receive electronic bills after online visits. Shanghai Sixth People’s Hospital is covered by social health insurance, issuing the first electronic receipt on June 23, 2020. For patients with conditions not catered to by internet hospitals, the system allows online doctor selection and appointment scheduling for offline visits. Meanwhile, internet hospitals can also provide health consultations to patients before offline clinic visits, providing advice without prescription issuance for medical safety considerations.

Based on the data we obtained, we constructed flowcharts for the offline and online hospital scenarios and summarized the treatment processes of the existing internet hospitals based on the doctor-patient interactions (Figure 3). Patients can receive consultations online before going to the hospital to enable the doctors to collect relevant health information and understand their current issues. If the patient needs to visit the hospital, they can undergo a more comprehensive and reliable consultation to assist in triaging. Next, patients can choose to register online or visit the hospital for an initial examination. After the first visit, patients can choose either online or offline options for evaluating disease prognosis, treatment effects, and further treatment. Furthermore, subsequent visits that require examination and laboratory tests need to be done offline. Therefore, the 2 areas are divided into online and offline options and colored in white and gray, respectively (Figure 3). Patients may also opt for in-home diagnosis and treatment options.

We also created an architecture chart of a typical approved internet hospital to demonstrate the fundamental structure of internet hospitals, detailing the full range of services offered, from appointment scheduling to ongoing health management (Figure 4). The system usually provides services to patient clients, doctor clients, and hospital clients. Patient clients mostly use messaging applications such as WeChat on mobile platforms, while doctor clients and hospital clients use more advanced functions and are mainly accessed through PCs. The services provided by approved internet hospitals can be divided into 5 categories: preoffline visits, online medical services, pharmacy services, payment-related services, and provision of health management information. In the preoffline visits module, patients can schedule appointments for offline clinics and request diagnostic tests (eg, COVID-19 tests) at home, which substantially lowers the risk of infection typically associated with community hospital settings. In addition, patients can use the online medical guidance system to determine which department they should visit. Approved internet hospitals offer a wide range of online medical services, including consultations, test report reviews, diagnosis, and prescription issuance. Due to the rapid development of logistics companies in China, internet hospitals can not only sell prescription drugs online but also provide door-to-door drug delivery services. In terms of payment-related services, patients can download invoices and pay bills directly through smartphones. An increasing number of approved internet hospitals have established partnerships with health insurance companies, allowing patients to use their insurance benefits online. Furthermore, because of the global reach of modern social media, internet hospitals can perform health education more effectively and assist patients in improving self-management practices. Some hospitals have specialized medical teams to support patients with chronic diseases, ensuring timely management of their conditions.

## Discussion

### Principal Findings

The focus of this study was to explore the development of patient-centric mobile medical services accessed through smartphones in the top 100 public hospitals in China. We also sought to identify the differences in providing patient-centric mobile health care services between approved internet hospitals and unapproved internet hospitals. The results demonstrated that almost all the top 100 hospitals (123/124) developed WeChat platforms or mobile medical services apps to offer mobile health care services to the public. Among the 124 hospitals, 60 (48.39%) were approved internet hospitals selected for analysis. Compared with unapproved internet hospitals, a greater number of approved internet hospitals provided online consultations, test reports, and chronic disease management. Furthermore, approved internet hospitals offering more than 6 mobile medical services were predominantly concentrated in Shanghai and Guangdong, consistent with the overall geographic distribution of hospitals.

By analyzing the geographic distribution of the top 100 hospitals offering more than 6 mobile medical services accessible through smartphones, we found significant diversity at the province level. Future research needs to delve into the factors driving this observed heterogeneity among hospitals in different Chinese provinces. Rigorous evaluations can also help the government formulate appropriate policies not only in China but also in other developing countries. In this study, we conducted a cross-sectional analysis to describe the characteristics of China’s mobile medical services accessed through smartphones and to assess their health care capacities. There are 6 categories of health services: appointment scheduling and registration, online medical guidance system and consultations, online prescriptions and drug delivery services, online test reports, online payment, and provision of health management. The appointment scheduling functions included online registration and examination tasks. Online consultations included online inquiries, access to the medical guidance system, and online referrals. Drug services included pharmaceutical information, prescriptions, and delivery. In addition, patients could access examination and laboratory test results online from many hospitals, although notifications of abnormal ancillary test results were not available from all hospitals. Health education primarily disseminated health information periodically and provided details on chronic disease management. Assistance regarding the management of chronic diseases is a new domain in mobile medical services. Only 6.45% of the hospitals had chronic disease management modules, given the requirement for experienced medical teams for monitoring and management. Recent advancements in mobile medical technology have shown promise in facilitating daily self-management of chronic diseases such as diabetes, lung diseases, cardiovascular disease, and mental health disorders remotely [21]. However, not all tasks in clinical care can be transferred from desktop computers to smartphones or tablets without process alterations. There is a pressing need for a process to implement, teach, supervise, and evaluate clinical mobile medical services as well as mobile device and app competencies, which may contribute to the lower popularity of these apps in most public hospitals [22-24]. Despite all these advantages of mobile medical services, it is clear that many core functions of offline hospitals cannot be replaced by remote health care. The most significant value of mobile medical services lies in promoting the transition from the traditional hospital-oriented model to a patient-centric model, empowering patients to become the primary drivers of the health care process.

Due to the rapidly growing population of the aged, the extension of life expectancy, and advances in clinical medicine, chronic disease management has become a considerable challenge for health care worldwide [25]. More and more individuals are grappling with multiple chronic conditions, and that usually requires multiple providers and consumes more medical services such as offline clinic visits and medications, which leads to higher health care spending [26]. According to NCD (noncommunicable disease) Countdown 2030, chronic diseases accounted for approximately 40.5 million of the 56.9 million deaths that occurred worldwide in 2016 [27].

In China, chronic diseases such as stroke, ischemic heart disease, lung cancer, and chronic obstructive pulmonary disease (COPD) are prevalent. Data indicate that stroke and ischemic heart disease were the primary causes of death and contributors to decreased disability-adjusted life years (DALYs) at the national level in China in 2017 [28]. The Chinese State Council has included the prevention and control of chronic diseases as an important part of the Healthy China 2030 Strategic Plan [29].

Furthermore, a 2020 WHO survey revealed that the ongoing COVID-19 pandemic had disrupted NCD services in 122 (77%) of the 159 countries surveyed [30]. The new concept of twin epidemics of COVID-19 and noncommunicable diseases highlights the disproportionate impact of COVID-19 on individuals with NCDs, exacerbating inequalities in the distribution of medical resources and resulting in dire consequences for people’s access to health care systems. This situation underscores the urgent necessity to invest in online chronic disease management as an integral component of primary health care and universal health coverage.

The limited provision of chronic disease management modules in top Chinese public hospitals may stem from the hierarchical medical system, which mandates such services to be primarily offered in lower-tier hospitals. Mobile technology has several potential advantages for providing actionable medical advice, but it also comes with its own limitations and potential associated problems [31,32]. Based on the advantages of mobile technology, it is sufficiently reliable and powerful to improve patient services and patient-physician relationships [2]. However, patients are concerned about the security of messaging on a mobile application, the complexity of portal design, the difficulty of following instructions, and the inability to understand the information presented in online educational resources. Older patients and children, in particular, are more likely to encounter challenges in using advanced technology than patients in other age groups. We suggest that providing patients with easy-to-follow tutorial videos before using the patient portal could help those who are less familiar with the technology to better understand its operation. It is somewhat surprising that there has been limited research investigating the efficacy and patient satisfaction of mobile medical services accessed through smartphones. The majority of existing studies have been conducted in surveys of small patient populations in a single hospital. In addition, no explicit protocol exists as to what kinds of diseases could be diagnosed or managed online. The efficacy of mobile medical services adoption and applications needs to be further explored.

### Limitations

This study has several limitations. First, it may not fully capture the evolving landscape of internet hospitals in China, especially considering the ongoing increase in their numbers during the COVID-19 pandemic. Second, given the cross-sectional nature of the study, any observed differences could only be considered correlational rather than causal. In addition, the absence of a validated questionnaire for assessing satisfaction with telehealth services poses a limitation. However, we plan to address these limitations in future studies by incorporating data from various perspectives, including those of doctors, patients, and investors.

### Conclusions

Mobile health care services not only improve the quality of health care services but also improve patient relationships with health care providers. Patient-centric mobile medical services accessed through smartphones primarily focus on online appointment scheduling, registration, health education, and online test reports. The most popular functions in the top 100 Chinese public hospitals are online consultations, prescriptions, medication delivery, medical guidance, and early-stage chronic disease management, indicating their potential to enhance primary health care services across China. Approved internet hospitals provided more patient-centric mobile medical services. Our findings indicate that these applications have the potential to be a multifunctional health management agent, and future research will investigate the validity, efficacy, and patient satisfaction in greater depth.

## Abbreviations

COPD: chronic obstructive pulmonary disease
DALY: disability-adjusted life year
NCD: noncommunicable disease
STEM: science and technology evaluation metric

## Data Availability

The datasets generated during and/or analyzed during this study are available from the corresponding author on reasonable request.
